# Health is wealth and documents are the currency: exploring the role of NGOs in facilitating healthcare access for undocumented migrants in the Netherlands – a qualitative study

**DOI:** 10.1186/s12939-025-02594-0

**Published:** 2025-08-11

**Authors:** Irsa R. M. Hanssen, Mara A. Yerkes

**Affiliations:** 1 Department of Interdisciplinary Social Science, Utrecht University, Utrecht, The Netherlands; 2 Centre for Social Development in Africa, University of Johannesburg, Johannesburg, South Africa; 3 Cluster of Excellence “The Politics of Inequality”, University of Konstanz, Konstanz, Germany

**Keywords:** Undocumented migrants, Nongovernmental organisations (NGOs), Healthcare access, Netherlands

## Abstract

**Background:**

Undocumented migrants (UMs) in the Netherlands face significant barriers to accessing healthcare despite legal entitlements to ‘necessary medical care.’ Non-governmental organisations (NGOs) play a critical role in facilitating healthcare access for UMs, yet empirical research on their contributions remains limited. This study aims to explore how NGOs perceive their role in facilitating healthcare access for UMs in the Netherlands and how these perceptions align with their actual practices.

**Methods:**

The research employs a qualitative exploratory design, conducting twelve semi-structured interviews with professionals from various NGOs supporting UMs in the Netherlands. Thematic analysis was used to identify key roles and strategies employed by these organisations.

**Results:**

The data revealed four main roles: (1) mediating, (2) educating, (3) advocating, and (4) delivering services. The findings suggest that while Dutch NGOs appear to share a common goal of facilitating healthcare access for UMs within the existing Dutch healthcare system, some of their strategies exist outside the regular system, thereby creating a parallel system. This parallel system subsequently results in a discrepancy between how NGOs perceive themselves (i.e., the role they *think* they should take) and the role they play in practice. Findings further suggest that the Dutch NGOs in this study face a humanitarian-equity dilemma, where limited resources and high pressure from UMs’ immediate needs lead them to prioritize direct assistance over addressing systemic issues. Consequently, many NGO strategies offer temporary solutions that help individual cases but fail to integrate UMs into the regular healthcare system sustainably.

**Conclusions:**

Dutch NGOs play a critical role in facilitating healthcare access for UMs in the Netherlands. Despite their invaluable efforts in addressing immediate needs, NGOs risk legitimizing a parallel system that allows the state to retreat from its welfare duties. To facilitate lasting change, NGOs should focus more on educational and advocacy roles and collaborate to reduce costs and enhance effectiveness. This strategic shift is essential for creating sustainable, inclusive health care solutions that ensure UMs are integrated into the regular healthcare system, thereby upholding the principles of equity and justice.

## Introduction

Access to quality health care is a human right [1]. Yet ensuring equitable access to health care in practice is challenging for many groups, particularly undocumented migrants (UMs) [2, 3]. Their absence of legal residency status and precarious living conditions make them particularly vulnerable [4]. The poorer life quality of UMs increases their susceptibility to health problems, exacerbated by barriers to healthcare access [5, 6]. Consequently, UMs tend to disregard minor health issues, potentially resulting in more severe health complications [4]. Ensuring healthcare access for these migrants is thus essential for sustainable and equitable community health and prevention efforts. Untreated health issues can increase marginalization, hinder integration, and exacerbate illnesses, whereas healthy migrants are better equipped to integrate and contribute to society [7].

The situation in the Netherlands clearly illustrates these challenges. The Dutch government has undertaken restrictive measures to control migration since the early 1990s and has recently upscaled efforts to discourage migration and discontinue services provided locally to UMs. The Dutch Linking Act (*Koppelingswet*), implemented in 1998 to discourage illegal residency, effectively excludes UMs from public services like healthcare insurance by linking a migrant’s legal status to welfare state benefits. Health insurance is mandatory for accessing healthcare services in the Netherlands. Although individuals without insurance can still register with a general practitioner (GP), the requirement to then pay for this care themselves is especially challenging for many undocumented and uninsured individuals [8].

Recent political climates in the Netherlands have been characterised by increasingly restrictive migration approaches. At the time of writing, the coalition that proclaimed to have “the strictest migration policy ever” has just fallen [9]. It remains unclear what this means for migration policies, but the demissionary cabinet could still follow through with plans to reduce funding for healthcare provided to uninsured patients by €40 million [10]. Although intended to primarily target unemployed migrant workers and not undocumented migrants specifically, the measure reflects a wider trend of exclusion within Dutch healthcare policy. Civil society and NGOs responded critically to the plans, describing the proposed changes as “inhumane” [11]. This context underscores the policy relevance of our study, which examines how UMs access healthcare amid increasingly restrictive institutional conditions.

In theory, the European Convention on Human Rights (ECHR) [12] should counter the structural inequities in healthcare access created by such restrictive policies, as it states that UMs are legally entitled to “necessary medical care” without incurring any costs themselves. To ensure compliance with the ECHR, an exception to the Dutch Linking Act ensures UMs access to all services covered under basic health insurance. Moreover, since 2009, healthcare providers can claim reimbursement from the Centraal Administratie Kantoor (CAK) for medical costs incurred from treating “uninsurable” patients and those unable to pay, often including UMs [13, 14]. But securing conditions to healthcare access in theory does not guarantee access in practice, leaving the health needs of many UMs unmet.

Data on healthcare access for UMs is fragmented, but evidence from 2012 suggests that almost one-third (29%) of the range of 23,000–58,000 UMs^1^ in the Netherlands did not receive the necessary medical care to which they were entitled [15]. Moreover, many UMs exclude themselves from healthcare services due to shame, lack of information, or fear of being deported [13, 16]. Across Europe, similar barriers impact access to healthcare services for UMs: legislative, financial, and administrative hurdles; absence of interpretation and cultural mediation services; unreliable information regarding the health conditions and histories of migrant patients; limited awareness of entitlements and accessible services; and inadequate organisation and coordination among healthcare services [17–19].

Nongovernmental organisations (NGOs; i.e., self-governing and not-for-profit entities geared towards improving the quality of life for disadvantaged individuals, that are often philanthropic and humanitarian in nature; [20, 21]) have become active in many European countries to help UMs overcome these barriers and to fill gaps in state-supported healthcare provision. They function as “right intermediaries”, offering guidance and practical support in accessing healthcare services [22] (pp. 601–604). Some organisations also provide medical services themselves [23, 24]. Despite the longstanding role of NGOs in welfare states, their role as welfare service providers is contested. A particular concern is that portraying NGOs as a solution to barriers in welfare state provision might obscure issues related to their involvement in such provision [25]. For healthcare services specifically, NGOs risk creating parallel and potentially inequitable healthcare structures alongside existing welfare state structures. Such developments can legitimize the state’s retreat from its welfare responsibilities [25, 26], thus inadvertently helping to maintain healthcare inequities.

This study contributes to the literature on ensuring equitable healthcare access by investigating the role of NGOs in facilitating access to healthcare services for UMs in the Netherlands. Despite the recognition that NGOs frequently fill gaps left by the state’s reluctance or inability to provide welfare services, there has been limited empirical study on the specific types of services these NGOs offer to immigrants ineligible for (certain) state-funded services, and how they deliver these services across various settings [24]. Previous studies in the Netherlands have addressed the health status of UMs and barriers to healthcare utilization, mainly focusing on the formal healthcare system [13, 15, 16]. However, there is a notable gap in understanding the *informal* mechanisms through which UMs access healthcare services facilitated by NGOs. This study aims to fill this gap by exploring the roles and services provided by NGOs in the Netherlands, providing insights into the role NGOs play in the healthcare landscape. Simultaneously, considering the lack of visibility of UMs and their resultant heightened vulnerability [27], this study aims to raise awareness of the precarious living situations of UMs and of the efforts of NGOs to improve these situations, contributing to greater healthcare equity.

## Background

NGOs serve as crucial mediating institutions between a government and its citizens, to protect individual freedom and enhance community accountability for addressing social issues [28]. NGOs may also emerge to fill the needs unmet by the welfare state, particularly for minorities or marginalized groups within society [29]. Within Dutch society, there is a strong history of NGOs playing an extensive role in the delivery of public (i.e., welfare state) services [30]. NGOs in the Netherlands also function as membership and advocacy organisations, empowering citizens to respond to the state’s retreat from its welfare obligations [31]. In particular, far-reaching decentralization of social service delivery in the Netherlands and hence increased responsibilities for municipalities [32], coupled with significant budget cuts, has led to an increasingly important role for NGOs as municipal governments explore alternative modes of service provision [33]. In this space of splintered responsibility for the distribution of public goods, NGOs have sought to support municipalities to “fill service gaps left vacant by the rollback of welfare provisions” [34, 35] (p.45). The financing of these NGOs differs significantly throughout the Netherlands, with smaller NGOs lacking structural funding and being primarily dependent on volunteers. Larger NGOs often have access to more structural funding (e.g., through the Dutch Postcode Lottery or government funding), while yet others rely on church-based financing or occasional funding from municipalities to deal with complex societal issues. In relation to healthcare access, although UMs who are socially embedded in support networks or NGOs are more likely to claim their health rights in practice [13], these “shadow networks” of service provision are contested [36](p.314) as noted above. Such parallel service provision could unintentionally perpetuate health inequities by legitimizing the retreat of the welfare state.

Given that migrant and ethnic minority populations often report poorer health compared to the majority host populations, considerable research has been devoted to examining their barriers to healthcare access [37]. Several studies conducted in the Nordic welfare states have identified factors including lower economic status, cultural differences, language proficiency, lack of education or knowledge of the system, and lack of culturally sensitive healthcare services as barriers to accessing healthcare among immigrant populations [38–40]. An additional barrier arising from a lack of education and knowledge of the system is the difficulty migrants face in navigating the healthcare system [41]. Related to linguistic and cultural barriers, many studies emphasize that a lack of interpreting services constitutes a significant barrier, often leading to suboptimal communication and consequently potentially inadequate care provision for immigrant populations [17, 42, 43]. In addition to the barriers faced by documented migrants, UMs encounter additional challenges related to their undocumented status, which often results in the underutilisation of healthcare services [44, 45]. Fear is a major barrier to healthcare access for UMs and includes fear of deportation and stigma, which often combine with an overall distrust in authorities [13, 16, 46, 47]. Another significant barrier unique to UMs is related to the reimbursement arrangements intended to enhance healthcare accessibility, such as the 2009 reimbursement of healthcare costs through the Dutch CAK. In practice, these arrangements often increase administrative complexity, which hinders healthcare access and provision [44, 48].

Given the unique barriers UMs encounter and the increasingly important role of NGOs in addressing barriers to healthcare access, greater attention is needed for their role in ensuring healthcare access to UMs. This qualitative study contributes to the literature by empirically exploring the role of NGOs in the Dutch welfare state system offering guaranteed healthcare access and decentralized service provision. We ask: *How do NGOs working with undocumented migrants in the Netherlands perceive their role in facilitating access to healthcare services*,* and how does this compare with their actual practices?*

## Methodology

### Study design

This study employed a qualitative research design to explore the role of NGOs in facilitating access to healthcare services for UMs in the Netherlands. Given the limited existing research on this topic, the study adopted an exploratory approach to gather insights into the perceptions and practices of NGOs. Semi-structured interviews were conducted with professionals working at Dutch NGOs who work for and with UMs. This form of interview is suitable for exploring complex social issues, such as information on the perceptions of NGOs on their role and practices [49]. This method was also flexible, allowing additional themes to emerge while maintaining the same structure across all interviews [50]. Given ethical considerations related to interviewing UMs directly, interviews were conducted exclusively with professionals representing NGOs.

### Participant sample and recruitment

Participant recruitment took part in three steps. First, relevant NGOs were identified. Foundation LOS, a national organisation facilitating exchange and collaboration among NGOs supporting UMs, maintains a list of these NGOs on their website, which served as an initial source for potential participants. Additionally, relevant NGOs were identified using *De Sociale Kaart*, a website listing organisations assisting homeless and undocumented individuals. Only NGOs whose websites explicitly mentioned addressing healthcare access for UMs were selected for study inclusion. After identifying suitable organisations, the second step involved the purposive sampling of a geographically diverse group of NGOs throughout the Netherlands, resulting in a total sample of 17 NGOs. Following, these NGOs were contacted via email and asked to distribute an information letter about the study, including the request for their members to participate. When a personal email address was available on the NGO’s website, it was utilized instead of the general email address, aiming to enhance response rate [51]. The initial response rate from the purposive sampling was 47% (8/17). Two NGOs declined due to capacity issues, six provided no explanation and one responded after the recruitment phase of the research had been passed. To secure a broader sample, an additional three organisations were recruited through snowball sampling, utilising the network of already participating NGOs [50]. The final sample consisted of 11 NGOs. A total of 12 semi-structured interviews were conducted, whereby 2 participants worked for the same NGO but were interviewed separately. Participants held various roles within the NGOs, including social workers, lobby specialists, and volunteers (see Appendix A for an overview and participant numbers referred to in the Findings). Despite the small sample size, rigorous purposive sampling took place to ensure a diverse group of participants [51]. Moreover, the final sample included a variety of organisations differing in size and scope, encompassing both regional and nationally active NGOs from various locations across the Netherlands. The variety in the sample was reflected in the breadth and depth of responses, which differed depending on participants’ roles, experiences, and expertise. Sampling and the interviews continued until no new information was provided, indicating that saturation had been achieved [52].

### Data collection

Interviews were conducted face-to-face (5) and online (via Microsoft Teams; 7), averaging 40 to 60 min each. The mixed-mode approach was chosen to accommodate participants’ schedules, allowing them the flexibility to choose an online format that was more convenient for them, thereby ensuring sufficient participation within the sample. A semi-structured interview guideline was developed using existing literature, initially focusing on the activities, strategies, and operational challenges encountered by NGOs attempting to secure healthcare access for UMs. Activities and strategies were understood as all actions undertaken by NGOs to facilitate healthcare access. For instance, participants were asked: “What does the organisation do to help overcome the barriers to accessing healthcare? Could you provide examples?”. Additional questions regarding collaboration with other NGOs and stakeholders within the healthcare system, including municipal governments and healthcare professionals, were incorporated following the first interview. For instance: “To what extent do you collaborate with other NGOs? Or other civil society organisations? Do you collaborate with the government? What does that collaboration look like? Could you provide an example?”. The full interview guide can be found in Appendix B. Interviews were audio-recorded and transcribed verbatim using GoodTape software, and personal identifiers were removed during pseudonymisation to ensure individual respondents could not be identified.

### Data analysis

Thematic analysis was conducted, a suitable method that allowed for the systematic identification and interpretation of patterns within the interview data [49]. Analysis consisted of both deductive (i.e., theory-driven) and inductive coding. An initial familiarisation with the data focused primarily on codes derived from the literature, such as barriers to healthcare access (e.g., language barriers, cultural differences, administrative complexity) and enablers of access, such as knowledge of healthcare rights and availability of culturally sensitive services. This initial familiarisation also led to the identification of several inductive themes worthy of further exploration. For instance, all interviewees talked about building a parallel system. However, they did so in distinct ways in relation to their views on the role of NGOs. Other key themes identified at this stage were related to the activities, strategies and collaborations of NGOs. These initial themes were further explored by defining and progressively updating sub-themes, and interpreting the data contained within each sub-theme. Lastly, key themes were inductively refined by going back to the raw data and establishing overarching themes. Coding and analysis were performed with qualitative software NVivo. An example of how data excerpts were assigned specific codes and inductively categorized into overarching themes is presented in Table 1.

**Table 1:** Coding process: excerpts, codes, and emerging themes

Data excerpts	Codes	Themes
“And therein [week], workshops are offered that also improve their access to healthcare. So, there are six different workshops, of which one is specifically focused on how to access healthcare. These workshops provide practical tips on this topic.” (P4) “And we also provide information through educational sessions (voorlichting). So once a month, we have a themed evening.” (P11)	Goal of work to improve agency Course to improve knowledge on rights	Education Role
“But the knowledge does trickle down. Even among general practitioners, through practices. Of course, more and more doctors are collaborating within practices, which also makes a difference. They also know how to reach out to us; if someone doesn’t know something, they call or email us regularly.” (P10) “When we say someone has no income, they truly have no income. I believe we have achieved that over time.” (P5)	Brand awareness (naamsbekendheid) NGO believed by authorities	Status

### Ethics

#### Ethical approval

was granted by the Faculty Ethics Review Board of the Faculty of Social and Behavioural Sciences at Utrecht University (FETC 24-0930). Informed consent was taken from the participants and their confidentiality maintained. Only researchers had access to the participants’ identifying information.

### Findings

Dutch NGOs appear to share a common goal of facilitating healthcare access for UMs living in the Netherlands. Generally, the preference is to do this by facilitating ‘regular’ access, that is, access within the existing Dutch healthcare system, as this respondent noted:

*“Of course*,* it is always preferable for someone to be part of the regular system. For someone to have a general practitioner in the neighborhood where they live. Where they can build a kind of trust relationship. Where they don’t have to explain everything about their legal situation every time. And where a general practitioner thinks along with them or refers them when necessary.” (P1)*

However, acknowledging the shortcomings of the Dutch system, several respondents also mention strategies for facilitating healthcare for UMs occurring outside of the regular system, thereby creating a parallel system. This was viewed as unsustainable in the long term.

Consequently, there seems to be a discrepancy between how NGOs perceive themselves (i.e., the role they *think* they should take) and the role they play in practice. Four different roles emerged through the interviews: (1) the mediating role; (2) the educating role; (3) the advocacy role; and (4) the service delivery role. Note that while this section analytically distinguishes four separate roles, in reality, these categories are conceptually less distinct and often overlap.

### The mediating role

The first prominent role identified is the role of NGOs in mediating between UMs and relevant organisations or individuals to facilitate healthcare access. Interviewees highlighted the networking strength of NGOs through various activities. These include referring UMs to GPs whom they know are open to working with UMs, facilitating conversations with less willing GPs, and connecting with other NGOs to exchange knowledge on best practices. Additionally, NGOs assist UMs in securing funding for more complex and expensive care. Below is a quote that illustrates the important role NGOs play in mediating communication between care professionals and UMs:

*“We often spend the whole day working on clarifying things for our own clients. Clients often don’t understand that they have been referred. So*,* we frequently have to talk with all those general practitioners to figure out what was actually said.” (P2)*

NGOs’ mediation role addresses the primary concern highlighted by all participants: the difficulty in finding healthcare providers willing to manage the additional administrative challenges of treating UMs. To fulfill this role, NGOs rely on a network of care professionals willing to assist UMs. This strategy is crucial for integrating UMs into the regular healthcare system. This can involve formal collaborations, such as networks of dentists in Amsterdam and The Hague who volunteer their services. A less formal strategy involves maintaining strong relationships with local GPs. NGOs emphasize the importance of sustaining these relationships, noting that having GPs or retired GPs as staff within the NGO enhances communication with other healthcare providers about UMs’ needs. This participant’s account illustrates the robust networking capabilities and sensitivity of NGOs in maintaining strong connections with care professionals who are willing to care for UMs:

*“But a healthcare provider*,* like our dentist or optician [that we collaborate with; (ed)]*,* shouldn’t have everyone from all over [city] sent their way. That would be too much for anyone. We’re not a very large organisation*,* so we generally don’t rely on them excessively. We also try not to send too many cases at once. Otherwise*,* you overwhelm them. If they’re doing it for free*,* you have to be considerate”. (P3)*

It is important to note that this network and connection with care professionals extends both within and outside the regular health system. Some healthcare professionals assist UMs within their own practice without officially registering them as patients, thereby still excluding UMs from the regular healthcare system.

### The educating role

The educating role of NGOs is twofold: education provided by NGOs directed at UMs and education and knowledge sharing with other organisations or healthcare professionals. The educational activities directed at UMs consist of things such as a WhatsApp helpdesk, informative websites, and walk-in hours where people can ask questions or get guidance in navigating the Dutch healthcare system. This guidance also included support with handling digital forms and online registrations, which NGOs identified as a relatively new and growing barrier for many UMs. Workshops and courses related to disease prevention were also mentioned. NGOs active in the same region also mentioned collaborative efforts to educate undocumented women on sexual and reproductive health. Additionally, NGOs organize workshops to educate UMs on their healthcare rights and how to claim them. The effort underlying most of these educational activities is to empower UMs by equipping them with information to assert their rights effectively, as illustrated by this quote:

*“And then there is a ten-week course*,* with one of those days really focused on access to medical care. We also do a sort of role play*,* like if you’re at a doctor’s office and they say no*,* I’m not going to help you*,* you’re not entitled to this. Then we really practice standing up for yourself.” (P4)*

Educational activities targeting care professionals and other stakeholders aim to inform these groups about the rights of UMs and the potential roles they can play in supporting UMs. The extent to which such educational efforts towards professionals are focused and structured appears to vary. For example, one NGO provides structured education through accredited e-learning modules on care provision for UMs. In most cases, however, NGOs adopt less formalized approaches, like explaining the reimbursement arrangement to care professionals. Such informal education and knowledge sharing occurs when professionals either refuse to help UMs due to payment concerns or when they independently seek clarification from NGOs.

### The advocacy role

In their advocacy role, NGOs aim to signal the issues faced by UMs in accessing healthcare, employing indirect strategies to inform public opinion and influence local and national policy. NGOs perceive advocacy as integral to their mission, as articulated by one participant: “*Our approach has always been both individual support and collective struggle to improve the lives of undocumented people.” (P5)*

Participants mentioned engagement in advocacy activities, but approaches varied among NGOs. These activities include publishing reports, organizing conferences, campaigning, and engaging in media outreach to raise awareness about pressing issues concerning UMs. Some NGOs also engage in lobbying efforts, such as sending complaints to the National Ombudsman. At the local level, some NGOs have regular meetings with the municipality to discuss issues and initiatives related to UMs’ healthcare access. NGOs also vary in their experiences of lobbying effectiveness. For example, one participant expressed frustration over perceived ineffectiveness in lobbying, mentioning uncooperative municipal officials. An NGO working throughout the Netherlands mentions that local lobbying effectiveness depends on the specific context and “*sometimes municipalities are quite willing to set up various initiatives or designate their own subsidy funds specifically for access to care for this group. Because of this*,* we see that local lobbying can be very effective.” (P6.1)*

### Service delivery role

A final role emerging from the interviews was the direct provision of healthcare services by NGOs to UMs. One NGO operates its own GP practice and has mobile healthcare units that visit various cities. These services often involve collaboration with other NGOs at their facilities. Within this role, it is clear that distinctions between NGO volunteers and staff working in public health services are often blurred. In many cases, the interviews revealed that the doctors providing services through NGOs are retired doctors and GPs or volunteers who devote part of their time to NGOs. In contrast to the mediating role of NGOs, where they facilitate healthcare access within the ‘regular’ system, in their service delivery role, healthcare is explicitly provided outside of the regular care system. Some NGOs we spoke to opt to not provide care directly but rather refer UMs to other NGOs that do, thus demonstrating an overlap between mediating and service provision roles. Participants emphasize that NGOs offering direct healthcare services guarantees ease of access for minor health issues but also express concerns about the quality of care for more serious health issues in comparison to the regular healthcare system. Additionally, some interviewees mentioned that NGOs taking on direct service delivery face high demand and are frequently overwhelmed. It is important to note that for most NGOs we spoke to, direct service provision was the lowest priority or even explicitly avoided due to concerns about perpetuating a parallel healthcare system for UMs. One NGO explained, for example, that by providing care: *“you end up only taking care of the people who come to you*,* and you shouldn’t want to build a parallel system to relieve the other system”. (P7)*

### Factors shaping the roles of ngos in access to healthcare for undocumented migrants

Alongside an exploration of the role of NGOs in securing access to healthcare for UMs, we also explored factors shaping the roles taken on by NGOs. Our findings suggest multiple factors are shaping which roles NGOs take on and when. A recurring theme in the interviews was the lack of resources, including time, money, and (volunteer) staff, which prevented NGOs from taking on certain roles. Resource constraints lead NGOs to prioritize direct assistance to UMs in the form of mediation (e.g., facilitating access) and education (e.g., informing UMs about their rights), thus helping UMs navigate the Dutch healthcare system. Many NGOs experience the needs of UMs as overwhelming and immediate, leading them to opt for this direct approach rather than engaging in more indirect methods such as lobbying or campaigning. NGOs also spoke about having to justify to funders and donors how these limited resources are used, which plays a role in what NGOs can do and how they do it:

*“I once suggested to a fund that […] there should be something to help professionals easily understand all the rights. She responded*,* ‘You don’t need to educate professionals*,* do you?’ So*,* it’s harder to get support for that than if you say*,* ‘I am literally helping someone.’” (P8)*.

A second factor shaping the roles NGOs take on is collaboration, which was seen as an important enabler for fulfilling all four roles. NGOs highlighted the importance of working with various actors within the healthcare system, including other NGOs within their region, but also collaboration across regions aimed at sharing successful strategies and addressing common challenges. Collaboration also enables smaller NGOs to take on roles they would otherwise be unable to take. Larger NGOs, often with more resources, support smaller NGOs by assisting in the development and maintenance of their programs. Such collaboration was specifically noted as an enabler for the advocacy role, where larger NGOs set up lobbies or campaigns joined by smaller NGOs in support of these efforts. However, collaboration was also seen to require both time and effort, both of which are often in short supply for NGOs with limited resources. The following quote illustrates how NGOs attempt to balance their limited resources while also trying to facilitate collaboration with other NGOs:

*“NGO [anonymous name] organizes a meeting once a month*,* I think [of NGOs at the national level; red.]. We always get an invitation*,* but we don’t attend very often because we’re quite busy. However*,* I still believe we should focus on it a bit more.*” *(P1)*.

A third factor that emerged in the interviews was the influence of status, where NGOs that are well-known or have a recognized status in their region were seen to have more influence in fulfilling certain roles. This influence was highlighted by several participants, who explained that when representatives of NGOs with recognized status contact healthcare professionals on behalf of UMs, particularly when professionals have initially refused to assist, cases are subsequently taken more seriously and the UMs are more likely to receive the care they need. Some NGOs even provide booklets or stamps for UMs to show they have been in contact with a high status NGO, facilitating healthcare access.

However, one participant’s account suggests that the ability of NGOs to secure care for UMs may be less about the NGO’s status and more about the privilege of native Dutch employees within the NGOs or the presence of discrimination and racism among healthcare professionals:

*“A while ago*,* for example*,* a woman at a midwife’s office was approached by the assistant three times during her appointment about not having health insurance and needing to pay. […] So*,* I said*,* you can just point out the CAK agreement [that regulates payment for healthcare services to UMs*,* red.]. But if they keep saying that three times*,* it helps to call the healthcare provider. And it’s really unfortunate*,* but then I*,* as a white Dutch person*,* say*,* there is a CAK arrangement. And she [the UM*,* red.] has probably already told you about it. Midwifery care is covered*,* so please help her. […] and then it’s settled*,* and no more questions or complaints are raised” (P1)*.

Additionally, interviewees explained that in their experience, they have often developed a sense of trust with UMs, who often refer each other to NGOs. Respondents mentioned that UMs frequently find it more comfortable to seek assistance from NGOs rather than from GPs or other official institutions. One participant mentioned that, *“[…]*,* there’s a bit more trust in organisations that operate outside the system because they think*,* yes*,* we remain a bit more out of sight*,* and that might be safer for us since we are undocumented.” (p.06.1)*.

Finally, the findings highlight broader, systemic issues within the Dutch healthcare system that shape the roles of NGOs in facilitating access to healthcare for UMS. Examples include the lack of available GPs and healthcare costs that exceed basic insurance coverage. One participant mentions that:

*“These are problems that affect the entire society. You often notice that people in vulnerable positions*,* such as undocumented migrants*,* are the hardest hit by the immense pressure on healthcare. So*,* I think it’s primarily the overall understaffing in healthcare that trickles down to the rest of society.” (P9)*.

These structural issues in the Dutch healthcare system are difficult for NGOs to address. As one participant explains:

*“[…] and that is very clear*,* for example*,* in GGZ (mental healthcare). We also receive a lot of questions and requests for help regarding that. But the waiting list is ridiculously long for everyone. So*,* we won’t be able to change anything for an undocumented person. No matter how dire the situation sometimes is.” (P6.2)*.

Some NGOs view these systemic healthcare issues as both challenges and opportunities, in particular for their advocacy role. They highlight issues such as dental care not being covered by basic insurance and the lack of language interpretation in medical settings, which also affect other marginalized groups in society, not just UMs. Consequently, these NGOs strategically align their lobbying and campaigning efforts around barriers shared by other socioeconomically disadvantaged or migrant communities, aiming to receive broader societal support for improving healthcare access.

## Discussion

Although UMs in the Netherlands are legally entitled to basic healthcare access, formal rights mean little unless they can be effectively realized. In practice, UMs often encounter significant barriers to healthcare access, exacerbating their vulnerability to health issues [5, 6]. In many European countries, NGOs have emerged to help UMs overcome these barriers and fill gaps in state healthcare provision. However, empirical studies remain limited. In countries like the Netherlands, where healthcare responsibilities are shared between the welfare state and municipal governments, and efforts to restrict migration and access to services for UMs are increasing, insights are needed to tackle health inequities.

To address this gap, this study aimed to explore how NGOs working with UMs in the Netherlands perceive their role in facilitating access to healthcare services, and how these perceptions compare with their actual practices. The findings suggest that while Dutch NGOs appear to share a common goal of facilitating healthcare access for UMs within the existing Dutch healthcare system, taking on roles of mediation, education, and advocacy, some of their strategies help to create and maintain a parallel system, particularly in their role of direct service provider. Consequently, there seems to be a discrepancy between how NGOs perceive themselves (i.e., the role they *think* they should take) and the role they play in practice.

Our qualitative exploration suggests that this discrepancy stems, in part, from systemic issues within the Dutch healthcare system that are challenging for NGOs to address. Systemic issues include the shortage and overburdening of GPs, who have been tasked with additional responsibilities previously managed by other entities [53]. Moreover, NGOs recalled difficulties in finding care professionals willing to assist UMs, citing administrative hurdles and discrimination as contributing factors. This finding is in line with discussions on the role of racism as a social determinant of migrants’ health and it acting as a crucial barrier to accessing health services [54]. Further research into racism within the Dutch healthcare system, specifically concerning UMs, would be beneficial in this regard. Such information could enhance their advocacy efforts, as NGOs have indicated that addressing broader societal issues that affect a wider demographic is more effective than concentrating their advocacy solely on issues that specifically target UMs. By framing their advocacy within the context of larger societal challenges, NGOs can build broader support for their work.

Our findings further identified varying priorities in the roles taken on by NGOs, reflecting their perception that they play a critical role in addressing immediate and urgent healthcare needs of UMs. Simultaneously, NGOs struggle to decide whether to invest in education and advocacy, with the aim of long-term systemic improvement or direct assistance to individuals in urgent need of care. Given their experience of the overwhelming and often urgent needs of UMs, NGOs often opt for the latter, focusing on direct aid and services rather than long-term systemic change. Moreover, the complexity of needs among UMs significantly challenges NGOs in their efforts to assist UMs in accessing healthcare. Respondents noted that many UMs often deal with multiple, interconnected problems, not limited to health, requiring care across various domains from a range of care providers. This, in turn can heighten the perceived urgency of these needs. This complexity highlighted by our respondents is reflected in studies of UMs, who are seen to have: “multiple, interconnected needs that span health and social issues and require different healthcare (e.g., mental healthcare or addiction care), social care (e.g., social benefits) and welfare services at the same time” [55]. Additionally, our exploratory findings indicate a potential shift towards increased direct aid and healthcare service delivery to UMs by NGOs in the Netherlands. Such direct service delivery is critiqued, however, given concerns about establishing a parallel system and about the quality of such care. Extant research would suggest the latter concern is justified, as evidence from multiple countries indicates that NGOs primarily reliant on volunteers are unable to provide the same quality of care as the regular medical system [56, 57]. Thus, although NGOs expressed not wanting to exacerbate the exclusion of UMs from the regular healthcare system, their direct provision of healthcare services and facilitation of connections to healthcare professionals outside the regular system contribute to this parallel system. This discrepancy between what NGOs think they should do or aim to do and their actions can be understood through Piccoli & Perna’s argument that in contexts where the universal right to healthcare is legally established but not enforced by public authorities, civil society organisations encounter a fundamental dilemma between humanitarianism and equity [25]. Within the context of NGOs, many of which are driven by a humanitarian belief in alleviating human suffering, there is a tension between wanting to provide emergency care as well as non-emergency care to maintain overall well-being. But in doing so, governments in universal health care countries may use such efforts to shirk responsibility for achieving health equity [25]. Our findings suggest NGOs in the Netherlands are dealing with this humanitarian-equity dilemma in their work in ensuring healthcare access for UMs.

### Limitations

Some important limitations apply to our study. As an exploratory qualitative study, our findings, while based on a purposive sample [52], are not representative. Since it aims to explore rather than quantify, however, it is an appropriate method. Such exploration is particularly apt for examining the role of NGOs, which is often overlooked in health system research focused on systemized care because NGOs function as part of the “shadow network” assisting UMs. Nonetheless, the findings may be limited as not all NGOs approached agreed to participate in the study. Moreover, despite a thorough process of identifying and purposively sampling NGOs working with UMs, some NGOs may not have been identified and thus not approached for participation. Despite these potential biases, this study includes a geographically diverse and meaningful sample of NGOs active at the national level and of varying size. The richness of the information provided and the fact that theoretical saturation was obtained suggest a robust analysis of the question central to this study [52].

### Implications and recommendations

Limitations aside, our exploratory study offers insights into an understudied topic with important scientific and health equity implications. In countries with similar or even more extensive policies restricting healthcare access to UMs [58], NGOs are also likely to play an important role and may similarly be struggling with the humanitarian-equity dilemma [23, 25]. This remains an important issue for future research.

Implications for NGOs include the need to consider their implicit yet inherently political role in working with UMs [25]. Through their role as mediator and direct provider, they: “become part of political processes that draw the boundaries of who is included in, and who is excluded from, public services” [25](p.13). This observation is especially relevant in the current Dutch political climate, where migration policies are becoming increasingly restrictive, and anti-migration rhetoric often dominates public discourse. NGOs that rely on government funding may face reduced financial support, further exposing how healthcare access for UMs is shaped by political processes. NGOs must therefore approach their role in this space with a heightened awareness of these political dynamics.

Additionally, for many NGOs, the current strategies employed serve as temporary solutions, addressing individual cases rather than sustainably integrating UMs into the regular healthcare system. It has been argued that if NGOs focus on their advocacy role, they can avoid contributing to the creation of a parallel system [25], shifting focus to lobbying, campaigning or litigation to convince public agencies to take over the tasks currently managed by NGOs that support a parallel system. In the current context of possible reduced funding and increasingly restrictive policies, NGOs’ advocacy role becomes even more crucial. As political developments threaten the financial resources of NGOs, particularly for those engaged in the costly task of providing direct healthcare services, maintaining these parallel healthcare structures will likely become unsustainable. If UMs become increasingly dependent on these parallel structures, their access to care is at greater risk when funding declines. This makes it all the more salient to focus on integrating UMs into the regular healthcare system, as this offers a more realistic, sustainable solution in the long term.

We also note that while advocacy for legal changes may grant UMs better healthcare access in theory [44], our study shows that legal access alone is insufficient. Our findings suggest that a more effective advocacy approach would be to address practical barriers to healthcare access. It is important to acknowledge, however, that advocacy efforts can be time-consuming and expensive, posing challenges for NGOs with limited resources. Increased collaboration may offer avenues forward. In fact, collaboration among NGOs seems even more essential in the face of shrinking resources and political resistance. By working together, NGOs can reduce costs and make advocacy efforts more attainable, particularly for smaller organisations. Successful examples of collaboration among NGOs demonstrate that such partnership can enhance the impact and sustainability of their efforts [59]. Secondly, Siegmann et al. suggest focusing on addressing health care professionals’ lack of knowledge about UMs’ entitlement to healthcare [60]. Several participants mentioned that they already educate care professionals informally. However, NGOs could enhance these efforts by collaborating. For instance, they could pool resources to promote the e-learning for care professionals developed by one of the participating NGOs. A final recommendation arises from a novel finding in this research, not directly linked to the primary research question but significant in its implications. Throughout the interviews, it became evident that a significant barrier to healthcare access for UMs is the digitalization of healthcare processes, a topic not yet addressed in the literature on barriers to access for UMs. For example, many GPs in the Netherlands require online forms for registration, which creates at least two issues. First, UMs able to access these forms often cannot complete them due to the requirement of an address. Second, digital forms presume access to the internet, electronic devices, and the necessary digital skills. Research on the digitalization of welfare services highlights inequalities in access to electronic devices and digital skills, which could lead to unequal access to services [61, 62]. Further research into digitalization barriers for UMs is crucial. It would provide essential evidence for NGOs to effectively strategize and advocate for inclusive healthcare policies and practices, ensuring equitable access for UMs.

## Conclusion

Undocumented migrants in the Netherlands face significant barriers to accessing healthcare despite their legal entitlements to “necessary medical care”. This research demonstrates that NGOs play a critical role in facilitating healthcare access for UMs. Despite their invaluable efforts in addressing immediate needs, NGOs risk legitimizing a parallel system that allows the state to retreat from its welfare duties. The Dutch NGOs in this study appear to face a humanitarian-equity dilemma, where limited resources and high pressure from UMs’ immediate needs lead them to prioritize direct assistance and health services over addressing systemic issues. Consequently, many NGO strategies offer temporary solutions that help individual cases but fail to sustainably integrate UMs into the regular healthcare system. To increase lasting change, NGOs could focus more on educational and advocacy roles and collaborate to reduce costs and enhance effectiveness. This strategic shift is essential for creating sustainable, equitable and just solutions that ensure UMs are included in the regular healthcare system.

## Appendix A

**Table:** Participant Roles

Role	Sample	Participant Codes
Volunteer	4	P5, P8, P10, P11
Projectmanager	3	P3, P7, P9
Social worker	2	P2, P4
Program officer	2	P1, P6.2
Lobby specialist	1	P6.1
**Total**	**12**	

## Appendix B

**About the Organization** (if limited information is available on the website)

- Can you tell me what X does and represents?
- When was it founded, and why?
- Why does your organization want to help UMs in relation to healthcare?
- What is the goal of your work with undocumented migrants?

**Barriers for Undocumented Migrants**

- What barriers does your organization observe that undocumented migrants face when accessing healthcare?

**Activities of the Organization**

- What are your activities specifically related to healthcare for undocumented people in the Netherlands? How do you help them? Can you provide some examples?
- What does your organization do to help overcome the previously mentioned barriers to accessing healthcare? Could you give some examples?
- What strategies does your organization use to help UMs access healthcare?
- Which stakeholders in this field does your organization focus on in its work?
- Does your organization select projects or activities based on unmet needs you observe, whether from your own organization or other organizations/agencies?

**The Role of the Organization/NGOs**

- Can you describe your organization’s role in helping undocumented people access healthcare?
- How do you see your organization’s role in relation to other NGOs in the Netherlands?
- How would you describe your organization’s role in comparison to other actors actively working for and with undocumented migrants?
- To what extent do you collaborate with other NGOs? Or with other civil society organizations? Do you collaborate with the government? What does that collaboration look like? Could you give an example?
- Could you explain why you think NGO initiatives arise in the healthcare sector for undocumented people?
- Can you explain why you think other actors do not carry out or provide the activities/services your organization performs?

**Challenges for the Organization**

- What are some challenges your organization faces in its work to provide undocumented migrants with access to healthcare?
- How does your organization address these challenges?

**Future**

- Can you describe how you envision your organization’s role in the future?

## Data Availability

The data and material from this study are not available to other researchers given the confidential nature of interviews. Corresponding code schemes and other study materials are, however, available from the authors upon reasonable request.

## Footnotes

1 Actual numbers of UMs may be much higher, but precise estimations are challenging due to the inherent institutional invisibility of this group given their undocumented status (15).

## Abbreviations

CAK: Centraal Administratie Kantoor
GP: General Practitioner
NGO: Non-governmental Organisation
UM: Undocumented Migrant
